# Predictors of lymph nodes posterior to the right recurrent laryngeal nerve metastasis in patients with papillary thyroid carcinoma: A retrospective study

**DOI:** 10.1097/MD.0000000000007908

**Published:** 2017-09-01

**Authors:** Jiru Yuan, Jinghua Li, Xiaoyi Chen, Zhenwei Zhong, Zhengbo Chen, Ying Yin, Jialin Du, Shuzhen Cong, Zeyu Wu

**Affiliations:** a Department of General Surgery; b Department of Pathological Medicine; c Department of Ultrasound, Guangdong General Hospital, Guangdong Academy of Medical Sciences, Guangzhou, Guangdong Province, China.

**Keywords:** central lymph node metastasis (CLNM), lymph nodes posterior to the right recurrent laryngeal nerve (LN-prRLN), papillary thyroid carcinoma (PTC)

## Abstract

The aim of this study was to identify the risk factors associated with lymph nodes posterior to the right recurrent laryngeal nerve (LN-prRLN) metastasis in patients with papillary thyroid carcinoma (PTC).

A total of 81 PTC patients who underwent total/near-total thyroidectomy with LN-prRLN dissection in the Department of General Surgery at Guangdong General Hospital between June 2015 and August 2016 were assessed retrospectively. The relations of LN-prRLN metastasis with clinicopathologic characteristics of PTC were analyzed by univariate and multivariate logistic regression.

The incidence of LN-prRLN metastasis in patients with PTC was 51.9% (42 of 81 cases). Univariate analysis showed that lateral LN metastasis (*P* = .005), VIa central LN metastasis (*P* = .000), pathologic tumor size (*P* = .000), capsular invasion (*P* = .002), and extrathyroidal invasion (*P* = .018) (*P* < .05) were significantly associated with the increased incidence of LN-prRLN metastasis in PTC. No significant correlations were found between LN-prRLN metastasis and other variables such as gender (*P* = .056), age (*P* = .448), clinical N stage (cN) (*P* = .063), tumor location (*P* = .336), tumor site (*P* = .909), right tumor existence (*P* = .793), and multifocality (*P* = .381). Multivariate logistic regression analysis revealed that VIa central LN metastasis (OR: 4.490, *P* = .027) was independent risk factors of LN-prRLN metastasis in patients with PTC.

LN-prRLN metastasis is often indentified in patients with PTC. VIa central LN metastasis is an independent predictors of LN-prRLN metastasis, which allow for selective LN-prRLN dissection in patients with PTC.

## 1 Introduction

Thyroid cancer is the most prevalent endocrine malignancy in China.^[1]^ It is divided into 4 histological subtypes: papillary thyroid carcinoma (PTC), follicular thyroid carcinoma (FTC), medullary thyroid carcinoma (MTC), and anaplastic thyroid (ATC). Papillary thyroid carcinoma (PTC) is the most common type of thyroid cancer, accounting for about 80% to 85% of all thyroid malignancies.^[2]^ PTC have an excellent prognosis, with a 10-year survival >91% and 15-year survival >88%.^[3,4]^ Lymph node metastases (LNM) has been reported to be associated with increased recurrence and comprised survival in patients with PTC.^[2,5]^ The central compartment is the most common site of lymph node metastases and have been found in 20% to 90% of patients with PTC.^[6–8]^ It has been reported that the central neck local regional recurrence accounts for 74% of all recurrent cases in PTC patients.^[9]^

**Figure 1:**
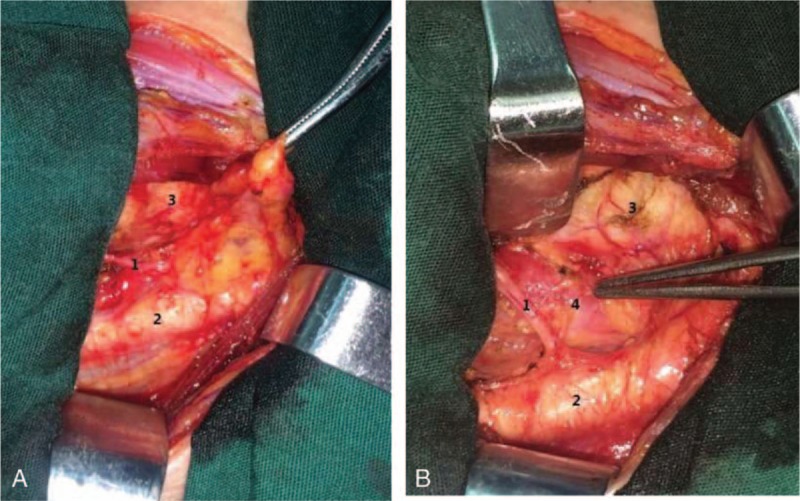
Surgical field during lymph nodes posterior to the right recurrent laryngeal nerve (LN-prRLN) dissection. (A) Before LN-prRLN dissection and (B) after LN-prRLN dissection: 1. Right recurrent laryngeal nerve; 2. Carotid artery; 3. Trachea; 4. Esophagus. LN-prRLN = lymph nodes posterior to the right recurrent laryngeal nerve.

Central lymph nodes could be dived into these parts: pretracheal nodes and paratracheal nodes, the Delphian nodes, and the perithyroidal nodes, including those along the recurrent laryngeal nerve (RLN).^[10]^ The left RLN and right RLN have differences in anatomical position. The left recurrent laryngeal nerve located closely next to the esophagus, and the right recurrent laryngeal nerve ascends through the fat tissue. Therefore, the right central neck lymph nodes were dived into posterior part and anterior part, but the left central neck lymph nodes only have anterior part. In this study, lymph nodes posterior to the right recurrent laryngeal nerve (LN-prRLN) were defined as VIb compartments, and lymph nodes anterior to the right or left recurrent laryngeal nerve were defined as VIa compartments. The LN-prRLN located in the area which is up to the inferior thyroid artery, down to the intersection of the common carotid artery and the tracheoesophageal sulcus, anterior to prevertebral fascia, and the lateral border is the medial border of the common carotid artery, and the medial border is the esophagus^[11,12]^ (Fig. 1). Complete central lymph nodes dissection (CLND) should remove VIa compartments and LN-prRLN (VIb compartments) simultaneously. However, LN-prRLN dissection was not accepted by most surgeons and often ignored during CLND. The main concern is that performing LN-prRLN dissection may increase the risk of RLN injury, resulting from exploring and rising RLN during the removal of the lymph nodes. For surgeons, it may be a better strategy to make an appropriate decision about the necessity of LN-prRLN dissection according to the likelihood of the presence of LN-prRLN metastases based on preoperative and intraoperative risk factors. Currently, few studies have been reported about the incidence and risk factors of LN-prRLN metastasis. Therefore, the aim of this study was to investigate the predictive factors that were associated with LN-prRLN metastases and assist surgeons in determining whether to perform selective LN-prRLN dissection in patients with PTC.

## 2 Materials and methods

A total of 81 PTC patients who underwent thyroidectomy with CLND in the Department of General Surgery at Guangdong General Hospital between June 2015 and August 2016 were retrospectively enrolled in this study. Preoperative assessment included ultrasonography (US), computed tomography (CT) scan, fluoro-18-deoxyglucose positron emission tomography (PET), chest x-ray, and measurement of thyroglobulin (Tg), thyroid stimulating hormone (TSH), and anti-Tg antibody levels. US was preoperatively performed to assess the lymph node status and confirm no lymph node involvement in all these patients. CT scan was used to observing suspicious invasion of the surrounding tissues or substernal thyroid cancers. PET scan was used in patients with suspected lung or bone metastases. Patients with previous thyroid or parathyroid surgery, previous neck surgery, family history of cancer, and history of neck radiation were excluded. All patients in our study underwent electronic laryngoscopy to see whether there was recurrent laryngeal nerve paralysis. The following information was collected from the medical records of the patients: gender, age, tumor size, bilaterality, multifocality, lymph node metastasis, capsular invasion, extrathyroidal invasion, TNM staging, recurrence stratification (RS), and postoperative complications. Follow-up was performed at 1 to 6 months after initial surgery. This study was approved by the institutional review board of Guangdong General Hospital. Informed consent was obtained from all individual participants included in the study.

In this study, there were 57 (70.4%) women and 24 (29.6%) men. The mean age was 42.5 ± 11.7 years, ranging from 17 to 61 years. There were 33 (40.7%) patients aged ≥45 years and 48 (59.3%) patients aged <45 years. US showed tumor diameter > 1 cm in 47 (58.0%) cases and tumor diameter ≤1 cm in 34 (42.0%) cases. All patients underwent bilateral CLND (including LN-prRLN) in addition to total/near-total thyroidectomy. In total, 12 (14.8%) patients underwent ipsilateral therapeutic lateral lymph nodes dissection and 1 (1.2%) patient underwent bilateral therapeutic lateral lymph nodes dissection. The central neck dissection included the comprehensive, compartment-oriented removal of all fibroadipose tissue between the trachea and carotid sheath from the hyoid bone superiorly to the upper mediastinum and the subclavian or innominate artery inferiorly. The delphian nodes and pretracheal lymph nodes should also be removed as part of such dissection.^[13,14]^ Subsequent radioactive iodine (RAI) remnant ablation therapy after initial surgery was recommended for the presence of multifocality, extrathyroidal invasion, and CLNM. Other patients took levothyroxin for TSH suppression and received regular follow-up with a physical examination.

The pathological examinations of surgical specimens were carefully performed by 3 pathologists with over 10 years of experience at our institution. All cases were confirmed as PTC using intraoperative frozen paraffin sections and postoperative paraffin sections. Single lobe diseases were found in 58 (71.6%) patients with PTC, both lobe diseases in 23 (28.4%) patients with PTC. Tumors were considered multifocal if ≧2 foci were found in 1 or both lobes. In the case of multifocal tumor, the largest dimension was used for statistical analysis. In this study, the tumor size cutoff of 1 cm was used for statistical analysis. For PTC, the pathological examination showed tumor diameter >1.0 cm in 32 (39.5%) cases and tumor diameter ≦1.0 cm in 49 (60.5%) cases. Pathological observation also revealed 23 (28.4%) cases with bilaterality, 31 (38.3%) cases with multifocality, 47 (58.0%) cases with central lymph node metastasis, 19 (23.5%) cases with capsular invasion, 6 (7.4%) cases with extrathyroidal invasion, and 3 (3.7%) cases with lymphovascular invasion. The mean number of VIa central lymph nodes was 4.27 ± 3.15 (ranging from 1 to 13) and the mean number of VIa central lymph nodes with disease was 1.41 ± 1.90. The mean number of LN-prRLN was 2.75 ± 2.15 (ranging from 1 to 10) and the mean number of LN-prRLN with disease was 0.69 ± 1.169. All patients were staged using the American Joint Committee on Cancer (AJCC) criteria^[15]^: 61 (75.3%) patients were in stage I, 16 (19.8%) in stage III, and 4 (4.9%) in stage IV. RS was according to the risk stratification of the American Thyroid Association.^[16]^ There were 28 (34.6%) patients with low RS, and 53 (65.4%)patients with intermediate RS.

### 2.1 Statistical analysis

Data collection was performed using Microsoft Excel. Statistical analysis was performed using SPSS 19.0 software. Data are presented as the means ± SD. Univariate analyses by the Pearson chi-square (*χ*2) test, Fisher's exact test, or 1-way ANOVA were performed to investigate the relationships between LN-prRLN metastasis and clinicopathological variables. Multivariate analysis was performed by binary logistic regression. *P* values <.05 were considered statistically significant.

## 3 Results

### 3.1 The incidence of LN-prRLN metastasis and postoperative complications

In this study, the incidence of LN-prRLN metastasis in patients with PTC was 38.3% (31 of 81 cases). Also, the incidence of VIa central LN metastasis in patients with PTC was 51.9% (42 of 81 cases). In addition, the incidence of both VIa central LN metastasis and LN-prRLN metastasis in patients with PTC was 32.1% (26 of 81 cases). LN-prRLN metastasis were found in 41.7% (5 of 12 cases) patients with only left lobe disease, 39.1% (18 of 46 cases) patients with only right lobe disease, and 34.8% (8 of 23 cases) patients with both lobes disease. There was no permanent hypoparathyroidism or permanent recurrent laryngeal nerve (RLN) palsy. Hypoparathyroidism is defined by low levels of calcium in the blood (<2.0 mmol/L) and/or low levels of PTH (<15 pg/mL).^[17,18]^ As for the level of postoperative parathyroid hormone, temporary hypoparathyroidism was found in 42 (51.9%) patients. However, only 17 (20.9%) patients had the symptoms of hypocalcemia. Transient recurrent laryngeal nerve palsy was found in 6 (7.4%) patients. Also, 2 (2.5%) patients had wound infection, which restored after conservative treatment. No Horner's syndrome, postoperative hemorrhage, and chyle leakage were diagnosed.

### 3.2 Correlations between LN-prRLN metastasis and clinicopathologic characteristics of PTC

Univariate analysis showed that LN-prRLN metastasis of patients with PTC was significantly associated with lateral LN metastasis (*P* = .005), VIa central LN metastasis (*P* = .000), pathologic tumor size (*P* = .000), capsular invasion (*P* = .002), and extrathyroidal invasion (*P* = .018) (*P* < .05). However, gender (*P* = .056), age (*P* = .448), clinical N stage (cN) (*P* = .063), tumor location (*P* = .336), tumor site (*P* = .909), right tumor existence (*P* = .793), and multifocality (*P* = .381) were not related with LN-prRLN metastasis (*P* > .05). In 12 patients with lateral LN metastasis, 9 (75.0%) patients were detected LN-prRLN metastasis, whereas in 69 patients with no lateral LN metastasis, only 22 (31.9%) patients were detected LN-prRLN metastasis. The difference between these 2 groups was statistically significant (*P* = .005). In addition, LN-prRLN metastasis were higher in patients with VIa central LN metastasis (26 of 42 cases, 61.9%), compared with patients with no VIa central LN metastasis (5 of 39 cases, 12.8%) (*P* = .000). LN-prRLN metastasis were less in patients with pathologic tumor size ≦1 cm (11 of 49 cases, 22.4%), compared with patients with pathologic tumor size >1 cm (20 of 35 cases, 62.5%) (*P* = .000). Also, LN-prRLN metastasis were less in patients with pathologic tumor size ≦2 cm (23 of 73 cases, 31.5%), compared with patients with pathologic tumor size >2 cm (8 of 8 cases, 100.0%) (*P* = .000). The rate of LN-prRLN metastasis with capsular invasion (13 of 19 cases, 68.4%) was higher than no capsular invasion (18 of 62 cases, 29.0%) (*P* = .002). Also, the incidence of LN-prRLN metastasis in patients with extrathyroidal invasion was higher (5 of 6 cases, 83.3%) (*P* = .018) (Table 1). On multivariate logistic regression, lateral LN metastasis (OR:2.400, *P* = .286), capsular invasion (OR:2.690, *P* = .165), pathologic tumor size (OR:2.685, *P* = .098), and extrathyroidal invasion (OR:2.402, *P* = .486) (*P* > 0.05). Only VIa central LN metastasis (OR: 4.490, *P* = .027) was independent predictors of LN-prRLN metastasis in patients with PTC (Table 2).

**Table 1:**
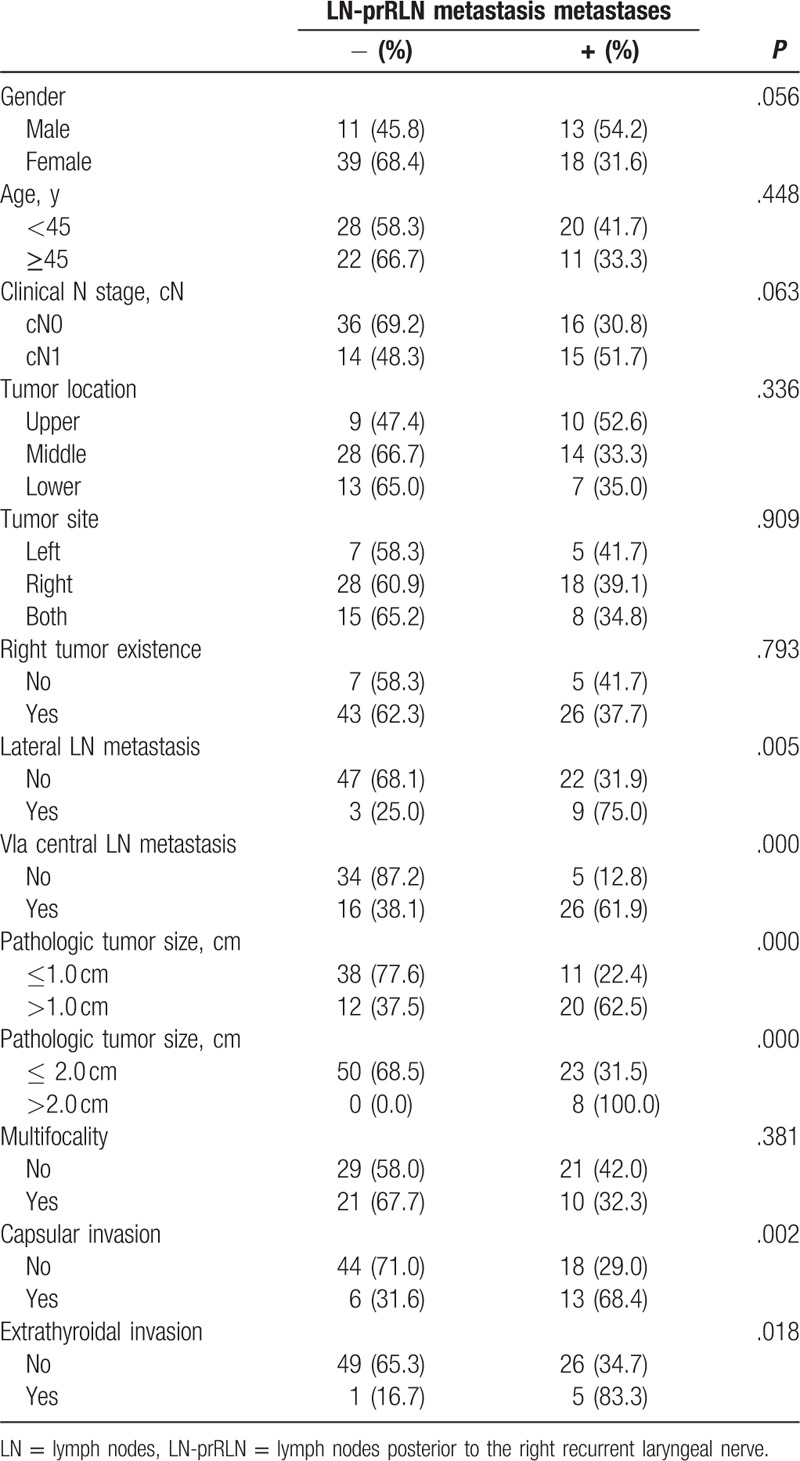
Univariate analysis of the correlations between LN-prRLN metastasis and clinicopathologic characteristics of PTC.

**Table 2:**
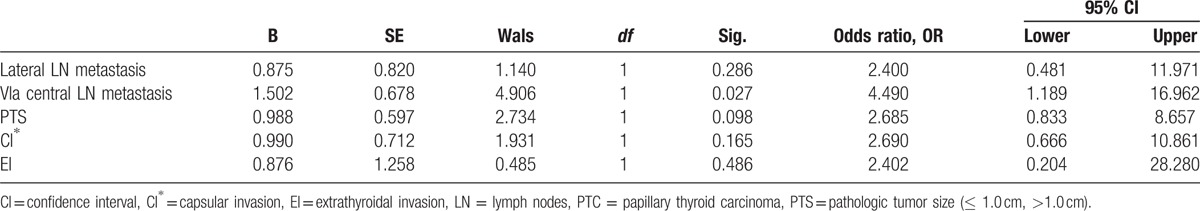
Multivariate analysis of the correlations between LN-prRLN metastasis and clinicopathologic characteristics of PTC.

## 4 Discussion

The guidelines of the American Thyroid Association recommend the use of preoperative ultrasonography to clinically detect relevant nodal disease and support the management of central lymph node dissection.^[19]^ But, the sensitivity of ultrasonography and computerized tomography to detect lymph node metastasis is low.^[20]^ The cervical lymph node metastasis is diagnosed in 20% to 90% of patients in PTC, and CLNM is well-associated with recurrence and overall survival.^[8]^ Therefore, it seems that to perform complete central neck dissection (CCND) in patients with PTC is necessary. However, it is controversial for performing complete central neck dissection which includes LN-prRLN dissection (VIb compartments). The main reason for the controversy around CCND may include balance of the potential benefits and the potential higher incidence of postoperative complications when LN-prRLN dissection was performed. So, it is important to identify the risk factors associated with LN-prRLN metastasis in PTC, which may assist surgeons in making the decision whether to perform selective LN-prRLN dissection. In addition, little has been reported about predictors of LN-prRLN metastasis in patients with PTC. Our study demonstrated that LN-prRLN metastasis of patients with PTC was significantly associated with lateral LN metastasis (*P* = .005), VIa central LN metastasis (*P* = .000), pathologic tumor size (*P* = .000), capsular invasion (*P* = .002), and extrathyroidal invasion (*P* = .018) (*P* < .05). Also, VIa central LN metastasis (OR: 4.490, *P* = .027) is an independent predictor of LN-prRLN metastasis, which allow for selective LN-prRLN dissection.

In present study, the incidence of CLNM was 58.0% (47 of 81 cases). However, the incidence of VIa compartments metastasis and LN-prRLN metastasis were 51.9% (42 of 81 cases) and 38.3% (31 of 81 cases), respectively. It should be noted that 5 patients (5 of 81 cases, 6.2%) had only LN-prRLN metastasis. In a retrospective study reported by Pinyi et al,^[21]^ 65 cases of LN-prRLN metastasis were detected among 286 cases of cN_0_PTC patients. Liu et al^[22]^ reported that 11.0% (16 of 145 cases) patients had LN-prRLN metastasis and Zhang et al^[23]^ reported that 27.2% (77 of 283 cases) PTC patients were found to have LN-prRLN metastasis. Considering the rate of LN-prRLN metastasis, we thought to complete central lymph nodes dissection including LN-prRLN is necessary. Grodski et al^[24]^ proposed that LN-prRLN dissection should be performed in CLND procedures as well. These results reminded that LN-prRLN dissection should be considered to be implemented during CLND.

In our study, patients with disease lobes including right lobe, both lobes, and left lobe were analyzed to identify risk factors for LN-prRLN metastasis. The right lobe and both lobes had disease that would be regarded as the right tumor existence, and the left lobe had disease would be regarded as no right tumor existence. A retrospective study reported that right lobe lesion is an independent risk factor for LN-prRLN metastasis in PTC patients.^[25]^ Lee et al indicated that the right lobe lesion was significantly associated with LN-prRLN metastasis in patients with PTC. However, multiple analysis revealed that the right lobe lesion was not an independent predictor.^[26]^ Therefore, there still exists controversy about the relations between right tumor existence and LN-prRLN metastasis. Larger sample data of prospective study may help us to get the right conclusion. In this study, the incidence of LN-prRLN metastasis in patients with right tumor existence was 37.7% (26 of 69 cases), lower than 41.7% (5 of 12 cases) in patients with no right tumor existence (only left lobe had disease). However, the difference was not statistically significant (*P* > .05). This result may be related to small number of samples. Interestingly, LN-prRLN metastasis was detected in 5 patients (5 of 12 cases, 41.7%) with only left lobe having disease. All these 5 patients were revealed multifocality and VIa central LN metastasis (Table 3). Zhang et al^[25]^ also reported that 2 cases with only left lobe having disease were diagnosed with LN-prRLN metastasis. Another study also reported 2 cases of left lobe tumor diagnosed with LN-prRLN metastasis.^[26]^ These studies indicated that we still should pay attention to the LN-prRLN metastasis in patients with only left lobe having disease.

**Table 3:**
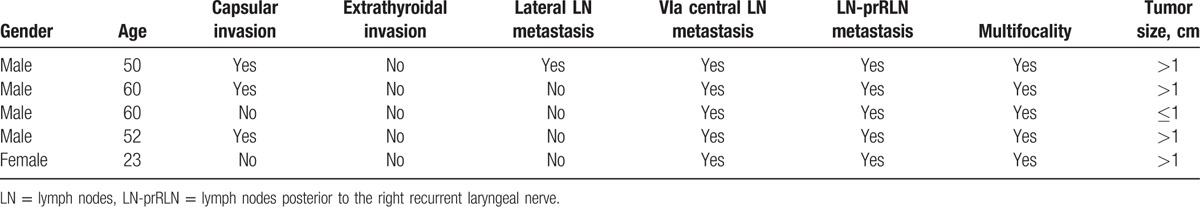
Clinicopathologic characteristics of LN-prRLN metastasis in patients with only left lobe had disease.

In this study, LN-prRLN metastasis was associated with VIa compartments central lymph nodes metastasis (*P* = .000). Multivariate logistic regression analysis confirmed that VIa compartments metastasis was an independent predictor (OR: 4.490, *P* = .027). Similarly, previous studies have reported that VIa lymph node metastases was an independent predictor of LN-prRLN metastase.^[25,26]^ Therefore, VIa compartments central lymph nodes status may help surgeons to determine whether to perform selective LN-prRLN dissection.

Previous studies have shown that the larger tumor size was the risk factor for the presence of LN-prRLN metastasis in patients with PTC.^[11,12,22,25–27]^ It has been reported that tumor size >1.0 cm was an independent predictor of LN-prRLN metastasis in patients with PTC.^[12,27]^ Ito et al^[28]^ reported that tumor size >2.0 cm was an important predictor of LN-prRLN metastases in patients with PTC in the right lobe. In the present study, LN-prRLN metastasis was more frequent in patients with tumor size >1 cm (20 of 32 cases, 62.5%), compared to patients with tumor size ≤1 cm (11 of 49 cases, 22.4%) (*P* = .000). We also found that all patients with tumor size >2.0 cm had LN-prRLN metastasis. However, multivariate logistic regression analysis revealed that tumor size was not an independent predictor of LN-prRLN metastases in patients with PTC. This may be explained by the small sample size in this study. Further, a larger sample study will be helpful to draw the right conclusion.

It was controversial about the relationship between extrathyroidal extension and LN-prRLN metastasis. Pinyi et al^[21]^ reported that extrathyroidal extension was an independent predictor. Liu et al^[22]^ had showed no correlations between extrathyroidal extension and LN-prRLN metastasis. In this study, extrathyroidal extension was associated with LN-prRLN metastasis in patients with PTC (*P* = .018), but not an independent predictor. We also found capsular invasion (*P* = .018) and lateral LN metastasis (*P* = .005) was significantly associated with LN-prRLN metastasis (*P* < .05). However, the differences disappeared in multivariate logistic regression analysis. Further randomized controlled multicenter studies will be helpful to draw the right conclusion. No significant correlations were found between LN-prRLN metastasis and other variables such as gender, age, clinical N stage, tumor location, tumor site, right tumor existence, and multifocality.

LN-prRLN was located in the central compartment deeply and was hard to expose. Transient hypoparathyroidism is the main complication of CLND (including LN-prRLN dissection). Lee et al^[26]^ reported that the incidences of transient hypoparathyroidism was 40.6% to 42.8%, and 3 (2.4%) patients had permanent hypoparathyroidism. Bae et al^[12]^ reported that 40 (10.8%) patients had hypocalcemia that required calcium supplementation. Another study^[25]^ indicated only 4 (1.6%) patients had permanent hypoparathyroidism. In the present study, the incidence of temporary hypoparathyroidism was 51.9%. The main cause of temporary hypoparathyroidism may be devascularization of parathyroid glands during dissection. Only 17 (20.9%) patients had the symptoms of hypocalcemia. This may be explained by the fact that preventive calcium was routinely supplemented in every patient since the day after surgery. No permanent hypoparathyroidism happened in this study, same to a prospective study.^[22]^ Temporary RLN injury is another important complication of CLND (including LN-prRLN dissection). In this study, there was no permanent RLN palsy, and transient RLN palsy was found in 6 (7.4%) patients who all recovered within 3 months after initial surgery. RLN palsy also found 1.0% to 7.0% in some studies,^[11,12,21,25]^ but all recovered within 6 months postoperatively. These results indicate that LN-prRLN dissection can be safely implemented during CLND.

In conclusion, LN-prRLN metastasis is often indentified in patients with PTC. The presence of lateral LN metastasis, VIa central LN metastasis, pathologic tumor size >1.0 cm, extrathyroidal invasion, and capsular invasion could assist surgeons in evaluating LN-prRLN status for PTC patients and considering the necessity of LN-prRLN dissection individually. VIa central LN metastasis is an independent predictors of LN-prRLN metastasis, which allow for selective LN-prRLN dissection. Considering the nerve injury that results from exploring and rising RLN during the removal of the LN-prRLN, we recommended performing LN-prRLN dissection in patients with 1 or more of these predictive factors. Also, LN-prRLN dissection must be performed by experienced and skilled surgeons.

The first limitation of this study was the fact that it was a study from a single center, and there might have been a selection bias. The second limitation of this study was that we could not evaluate the effect of LN-prRLN dissection on cancer-specific survival and recurrence rate of patients with PTC because the follow-up time was relatively short in this study.
